# Retraction: Consumption of pomegranates improves synaptic function in a transgenic mice model of Alzheimer’s disease

**DOI:** 10.18632/oncotarget.28773

**Published:** 2025-10-16

**Authors:** Nady Braidy, Musthafa Mohamed Essa, Anne Poljak, Subash Selvaraju, Samir Al-Adawi, Thamilarasan Manivasagm, Arokiasamy Justin Thenmozhi, Lezanne Ooi, Perminder Sachdev, Gilles J. Guillemin

**Affiliations:** ^1^Centre for Healthy Brain Ageing, School of Psychiatry, Faculty of Medicine, University of New South Wales, Sydney, Australia; ^2^Department of Food Science and Nutrition, College of Agricultural and Marine Sciences, Sultan Qaboos University, Al Khoudh, Oman; ^3^Ageing and Dementia Research Group, Sultan Qaboos University, Al Khoudh, Oman; ^4^College of Medicine and Health Sciences, Sultan Qaboos University, Al Khoudh, Oman; ^5^Department of Biochemistry and Biotechnology, Annamalai University, Tamil Nadu, India; ^6^Illawarra Health and Medical Research Institute, University of Wollongong, NSW, Australia; ^7^Neuropsychiatric Institute, The Prince of Wales Hospital, Sydney, Australia; ^8^Neuroinflammation Group, MND and Neurodegenerative Diseases Research Centre, Macquarie University, NSW, Australia; ^*^These authors have contributed equally to this work

**This article has been retracted:** Our Image Forensics analysis revealed Western blot image duplications in Figures 1A, 3A, 4A, and 5A. Specifically, some bands in different Western blots in Figure 1A appear to be the same, Synaptophysin blot is duplicated as Bcl1 blot in Figure 3A, b-actin blot is duplicated in Figure 4A as Akt blot and p-CaMKIIα bands are duplicated in APP blot in Figure 5A. In Figure 4A the b-actin bands were found in b-actin blot in Figure 5A. We also identified an external duplication where the SNAP25 blot in Figure 1A was reproduced as the GAPDH blot in Figure 5B of an unrelated paper published later [1]. The corresponding author Professor Guillemin has acknowledged these duplications and requested the retraction if the first author, Dr. Braidy, who performed all the Western blots in his laboratory at UNSW, could not provide supporting materials. Furthermore, both universities, where the research was conducted, initiated their own investigations. Macquarie University concluded that Professor Guillemin’s contributions to the paper did not meet their authorship threshold according to the Macquarie University Code for the Responsible Conduct of Research and requested his name be removed. The second request to withdraw authorship was received from Professor Perminder Sachdev, who stated that his “involvement in this paper was restricted to commenting on a near final version of the draft from a clinical perspective, upon request by Dr Braidy, first author.” The UNSW Conduct & Integrity Office also began an investigation but has not yet provided an update or responded to the journal’s inquiries. Consequently, based on the findings of multiple WB duplications, the absence of supporting data from the first author, Dr Braidy, and issues with authorships, the editorial decision has been made to retract this paper. The corresponding author Dr. Guillemin agreed with the retraction, but the rest of the authors either did not respond directly or could not be reached.

Original article: Oncotarget. 2016; 7:64589–64604. 64589-64604. https://doi.org/10.18632/oncotarget.10905
